# Measles Outbreak Investigation in Aneded District, Northwest Ethiopia: A Case-Control Study

**DOI:** 10.1007/s44197-024-00279-5

**Published:** 2024-08-01

**Authors:** Mengistie Kassahun Tariku, Abebe Habtamu Belete, Daniel Tarekegn Worede, Bantayehu Addis Tegegne, Simachew Animen Bante, Sewnet Wongiel Misikir

**Affiliations:** 1 Department of Public Health, College of Health Science, Debre Markos University, Debre Markos, 269 Ethiopia; 2 Department of Pharmacy, College of Health Science, Debre Markos University, Debre Markos, Ethiopia; 3 Department of Midwifery, College of Medicine and Health Science, Bahir Dar University, Bahir Dar, 79 Ethiopia; 4 Department of medical laboratory technology, Felege Hiwot Comprehensive Specialized Hospital, Bahir Dar, 680 Ethiopia

**Keywords:** Aneded, Ethiopia, Households head attitude, Measles, Outbreak

## Abstract

**Background:**

Between 2000 and 2018, global measles deaths decreased by 73%, but the disease remains prevalent in many developing countries, especially in Africa and Asia. Although Ethiopia was attempting to eliminate the measles, it still ranks fourth in the world in terms of the number of cases. The aim of the investigation was to describe the outbreak and identify its determinants in the Aneded district.

**Methods:**

Between March 3, 2020, and April 2, 2020, the 89 patients and 178 controls participated in a case-control study. Data were gathered by means of in-person interviews with household leaders. The attack and case fatality rates were determined. In multivariable logistic regression analysis, variables having a p-value of less than 0.05 were considered statistically significant cut-off points.

**Results:**

An investigation was conducted on a total of 89 measles cases, with 3 deaths and 178 controls. In total, there were 1.65 attacks per 1000 people, or 3.4% of the case fatality rate. There were 155 days of outbreak duration. The disease was significantly associated with being female [adjusted odds ratios (AOR) = 2.66; 95% confidence interval (CI) = 1.38–5.11], under 5 years old [AOR = 7.24; 95% CI = 2.58–20.31], positive in attitude [AOR = 0.22; 95% CI = 0.11–0.42], and having a contact history [AOR = 3.19; 95% CI = 1.67–6.10].

**Conclusion:**

The measles outbreak, with its higher attack and case fatality rate, has been influenced by factors like household attitudes, age, sex, contact and travel history and needs to be reduced through early detection, active surveillance, and fostering favorable attitudes towards disease prevention and control.

## Introduction

Measles is a highly communicable disease with a 90% chance of infection and a severe respiratory viral disease that can be prevented through effective vaccination [1, 2]. It is characterized by fever, malaise, cough, runny nose, conjunctivitis, and maculopapular rash and can be transmitted through aerosol, droplets, or direct contact with infected individuals [3–5].

Severe measles is more common in malnourished young children, particularly those with vitamin A deficiency or weakened immune systems, leading to severe complications like blindness, encephalitis, diarrhea, and pneumonia [3, 5, 6]. Measles complications occur in 30% of cases and are more common in children younger than 5 years, adults older than 20 years, pregnant women, and immune-compromised people [3, 6].

Routine measles vaccination for children, combined with mass immunization campaigns in countries with low routine coverage, are key public health strategies to reduce global measles deaths [3]. While vaccination has radically reduced global measles deaths—a 73% drop between 2000 and 2018 worldwide—measles is still common in many developing countries, particularly in parts of Africa and Asia. Globally, more than 413,308 confirmed cases and 140,000 deaths were reported to the WHO in 2018 [3, 7]. The majority (more than 95%) of measles deaths occur in countries with low per capita incomes and weak health infrastructures [3]. Even if the Africa Region, as well as Ethiopia, were working towards measles elimination by 2020 [8], Ethiopia is still the 4th -top country in the world in the burden of measles cases [9].

Since 1980, national immunization programs have provided vaccination services at health facilities and outreach sites, now recommending a measles vaccination dose at 9 months of age [8]. In 2019, Ethiopia introduced the measles vaccine second dose (MCV2) vaccination into the routine immunization program at 15 months of age [10]. Despite these efforts, in Ethiopia, a recurrent measles outbreak has occurred [11]. The attitude of the rural community toward the causes of measles shows that both natural and supernatural forces are mentioned as causes of the disease [11].

The recurrent measles outbreak was caused by low sub-national routine measles coverage, poor nutritional conditions (e.g., low vitamin A supplementation coverage), measles case handling, the accumulation of unvaccinated children in densely populated areas, and the seasonal hot weather between December and April [12].

A suspected measles outbreak occurs when five or more cases are reported per 100,000 population in a specific area, while a confirmed outbreak involves three or more laboratory-confirmed cases per 100,000 population (9).

Outbreaks occur when susceptible individuals exceed critical thresholds for transmission. Not all children receive protection from vaccinations; 15% of those vaccinated at nine months and 5% of those vaccinated at twelve months fail to immunize (3).

The administrative vaccination coverage of the Aneded district was greater than 100%. Except for 3 health posts, all health posts had no refrigerators. In the district, the most common public health emergency-targeted diseases are measles, epidemic typhus, typhoid fever, malaria, dysentery, and scabies. In 2020, malaria and measles occurred in an outbreak with a total of 1756 and 89 cases, respectively [13].

A measles outbreak investigation involves identifying and confirming suspected outbreaks through immediate and effective procedures to implement measures for control (1, 2). Measuring the epidemic’s scope, looking for more cases, locating the population at risk and the outbreak’s source, and implementing prompt case management to lower death and morbidity rates and stop future outbreaks are the goals of measles outbreak investigations (1–3). As soon as a suspected measles outbreak is reported, an investigation into it is launched within three hours (12). The early detection, reaction, and investigation of an outbreak can significantly reduce the risk of disease, death, and outbreak spread (3). This is a crucial step towards the complete elimination of measles. (13). Despite numerous investigations into measles outbreaks in Ethiopia, there is a lack of comprehensive data on the attitudes of caregivers and household heads towards disease prevention and control [14]. This investigation aimed to describe an outbreak in Ethiopia’s rural areas in terms of person, place, and time and identify determinants.

## Methods

### Study Period and Area

**Fig. 1:**
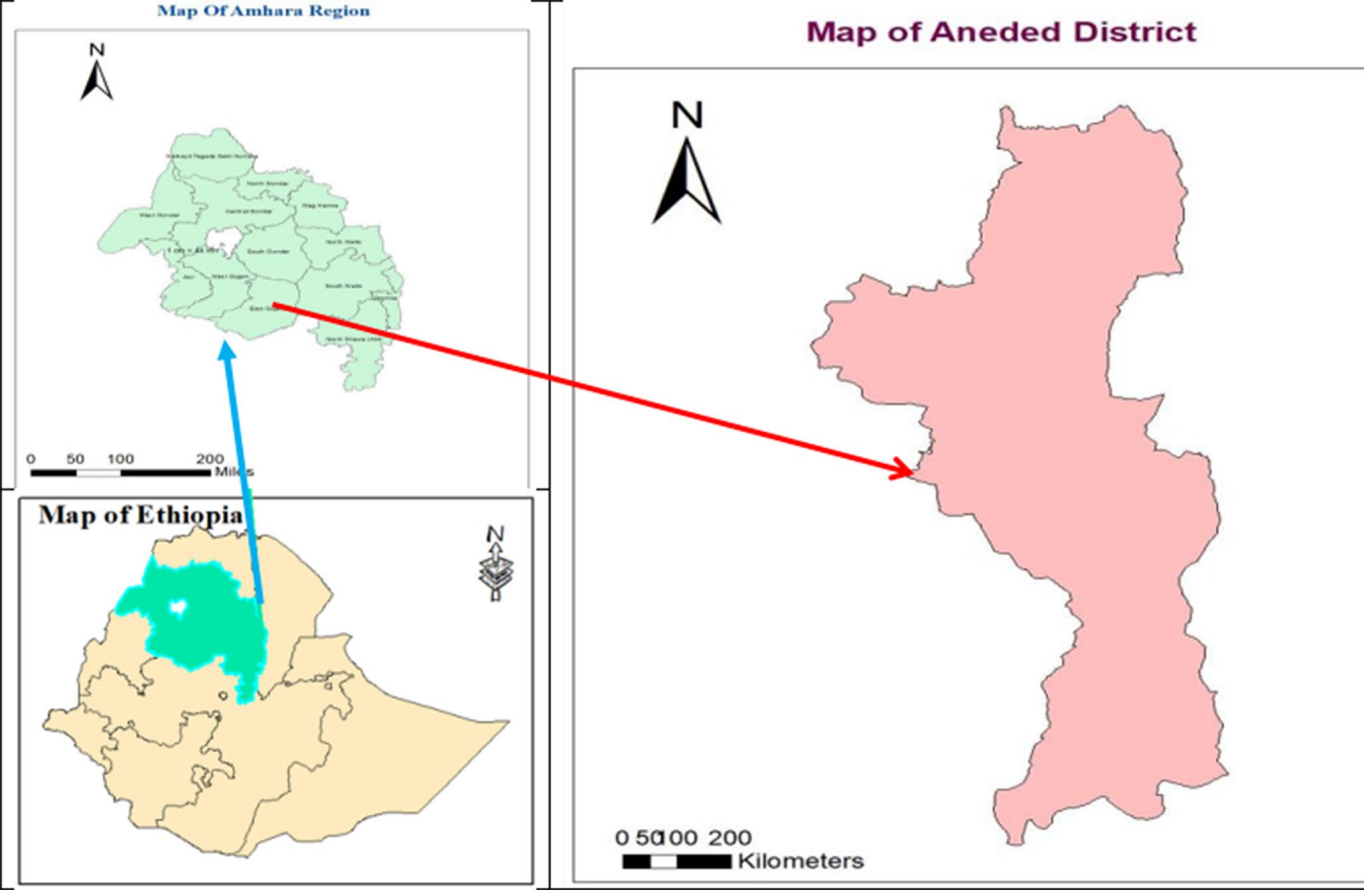
Map of Aneded District from Ethiopia and Amhara regional state, Ethiopia, 2020

The investigation was conducted from March 3, 2020, to April 2, 2020, in the Aneded district. The Aneded district is one of the districts in the East Gojjame Zone of the Amhara region, northwest Ethiopia (Fig. 1). It is bordered by the Gozamen district to the north, northwest, and west; the Baso Liben district to the southwest; the Awable district to the east, northeast, and southeast; and the Oromia region to the south. It is also 18 km (Km) away from Debre Markos, the capital city of East Gojjame, 285 km from Bahir Dar, the capital city of the Amhara region, and 267 km from Addis Ababa, the capital city of Ethiopia. Based on the 2007 population projection, there were a total of 109,110 populations: the total population of the district was disaggregated in terms of sex, age and Keble. and about 14,773 were under-5-year-old children [15]. The district has a population density of 319 inhabitants per square kilometer (830/sq. mill). The district had 19 rural kebeles (the smallest administrative units), which each owned a health post served by 38 health extension workers, 1 urban kebele, which was served by 1 health extension worker, and 4 health centers with a total of 70 health professionals and 60 supportive staff [13].

### Sample Size and Study Design

Using 89 cases and 178 controls, a 1:2 ratio and an unmatched case-control study were used. Using Epi Info version 7, we determined the sample size. In order to establish whether there was a significant association between the case status and vaccination status, the sample size was taken into account. The assumption of the predicted prevalence of non-vaccinated people at alpha 0.05 was 82.8% and 48.3% among cases and controls, respectively [15]. The calculated sample size could be 78 cases and 156 controls. By adding a 10% non-response rate, the sample size could be 86 cases and 172 controls. During the outbreak investigation, about 89 cases were identified. Then, there was no need for sampling from 89 cases. Therefore, the total sample size was 89 cases and 178 controls. Any person who had a fever, maculopapular rash, cough, conjunctivitis (red-eye), or coryza (runny nose) was included as a case, while any person who had none of the above-mentioned signs and symptoms was considered a control. By using the lottery method of simple random sampling, all eligible controls (neighbors or family members of cases who did not have fever, non-vesicular widespread rash, or cough) were chosen. People with a history of measles in the past, both cases and controls, were excluded.

### Case Definitions

Anyone with a fever and a widespread maculopapular (non-vesicular) rash together with one of the following is considered to be **a suspected case**: Cough, coryza, conjunctivitis (red eyes); or any individual that a medical professional suspects may have the measles. **A confirmed case** is a suspected case that has been linked to verified cases in an outbreak by epidemiology or laboratory confirmation (positive IgM antibodies). Any case that satisfies the criteria for a suspect case and is connected to a laboratory-confirmed case is considered epidemiologically linked. Clinically confirmed case: a case that does not have laboratory confirmation but nonetheless fits the definition of a suspect case and that a practitioner believes to be measles.

Measles deaths are defined as those that happen within 30 days of the rash starting and aren’t caused by any other unrelated factors (such as trauma or chronic illness). When five or more suspected cases of measles are documented in a single month per 100,000 residents of a certain geographic area, it is considered a suspected outbreak of the disease. A confirmed measles outbreak is characterized by the incidence of three or more confirmed cases in a month at a health facility or district (about 100,000 people in the catchment area), at least two of which must be laboratory-confirmed and IgM positive. Excluded from the control group were those who previously showed measles symptoms and signs [1, 4, 6].

### Outcome and Independent Variables

Having measles (case) or not having measles (control) was the outcome variable. Socio-demographic variables (age, sex, marital status, educational level, occupational status, residence, and household size), vaccination status, time taken to reach the nearest health facility, history travel 7–18 days prior to the onset of illness, contact history with measles cases, attitude, and knowledge were independent variables.

### Data Collection Procedure

A standard questionnaire was developed after reviewing different kinds of literature. The questionnaire consisted of socio-demographics, vaccination history, travel history, and distance from the nearest health facilities. Eight attitude-related questions were reliable and valid because they were adapted from literature [16, 17]. Twelve knowledge-related questions were reliable and have been validated for use in different settings [16–19]. Statistical package for social science (SPSS) was used to compute the questions pertaining to knowledge and attitude. The knowledge-related questions consisted of yes-or-no answers. If the answer was correct, we assigned 1 point, and for an incorrect answer, we gave 0 points. Knowledge-related questions were added, and then the median was performed. If the household heads answered above the median, they were assigned as knowledgeable. The attitude of household heads was measured with a Likert scale. Household heads who had attitude scores below the median were classified as having a negative (unfavorable) attitude. The internal consistency of the questionnaire was assessed by focusing on the inter-item correlations within the questionnaire, showing how well the contents are close-fitting together. This was done using Cronbach’s alpha (α). The value of Cronbach’s α was 0.80 [20]. House-to-house active case searching was conducted by eight trained diploma nurses to include all cases in the list. The face-to-face interview technique was used to collect data from households or caregivers.

### Data Quality Control

The English version of the questionnaire was translated into the local language (Amharic) for data collection. Then the Amharic version of the questionnaire was translated back into the English language to check its consistency. Two-day training about the objective of the study and data collection was provided for data collectors and supervisors. The collected data was checked for completeness and consistency by supervisors on a daily basis.

### Data Processing

After the data were coded and imported into Epidata version 3.1, they were exported to SPSS version 23 for further examination. Descriptive statistics like attack rate and case fatality rate were calculated prior to binary logistic regression analysis.

In bivariable binary logistic regression analysis, predictor variables with a P-value of < 0.2 were carefully chosen for multivariable binary logistic regression analysis. A multivariable binary logistic regression analysis was employed. Each predictor variable with a p-value of less than 0.05 was taken as a statistically significant cut-off point in multivariable binary logistic regression analysis.

## Results

### Descriptive Epidemiology

#### Characteristics of Study Participants

A total of 89 measles cases, with 3 deaths and 178 controls, were investigated. More than three-fifths (62.9%) of cases were female, while more than half (97.5%) of controls were male. The median age of cases and controls was 12 years [interquartile range (IQR) = 4–19.5] years and 23 years [IQR = 10.8–30.0] years, respectively. In terms of educational backgrounds, 37 (41.6%) of cases and 78 (43.8%) of controls couldn’t read and write. Sixty-nine (77.5%) of the household heads of the cases and 114 (64.0%) of the controls were not knowledgeable about measles disease transmission and prevention. Regarding the attitude of the household heads, 52 (58.4%) of cases had no favorable attitude toward measles disease prevention and control, while 139 (78.1%) controls had a favorable attitude. Almost three-fourths (69, or 77.5%) of cases and 126 (70.7%) of controls were not vaccinated. The majority, 60 (67.4%) of cases and 162 (91.0%) of controls, had no history of travel within 7–18 days before the outbreak. Forty-eight (53.9%) of cases and 60 (33.7%) of controls were traveling for more than an hour to reach the nearest health facility. More than half (47, 52.8%) of cases had a contact history with measles cases, whereas 133 (74.7%) of controls had no contact history with measles cases (Table 1).

**Table 1:** Characteristics of study participants during measles outbreak investigation in Aneded district, northwest Ethiopia, 2020

Variables		Case	Control
		N(%)	N(%)
Sex	Female	56(62.9)	97(54.5)
	Male	33(37.1)	81(45.5)
Age	< 5	26(29.2)	9(5.1)
	5–14	25(28.1)	59(33.1)
	≥ 15	38(42.7)	110(61.8)
Residence	Rural	86(96.6)	172(96.6)
	Urban	3(3.4)	6(3.4)
Educational status of study participants	Not eligible	10(11.2)	6(3.4)
	Unable to read and write	37(41.6)	78(43.8)
	Read and write but have no formal education	23(25.8)	55(30.9)
	Primary	19(21.3)	39(21.9)
Occupation status	Not eligible	32(36.0)	33(18.5)
	Student	16(18.0)	92(51.7)
	Farmer	41(46.1)	53(29.3)
Marital status	Not eligible	56(62.9%)	65(36.5)
	Single	21(23.6)	16(9.0)
	Married	12(13.5)	97(54.5)
Number of people living	< 5	45(50.6)	98(55.1)
	≥ 5	44(49.4)	88(44.9)
Attitude	Favorable	37(41.6)	139(78.1)
	Unfavorable	52(58.4)	39(21.9)
Knowledgeable	Yes	20(22.5)	64(36.0)
	No	69(77.5)	114(64.0)
Vaccination status	Yes	20(22.5)	52(29.2)
	No	69(77.5)	126(70.8)
Time taken to reach the nearest health facility	1 h	27(30.3)	63(35.4)
	Less 1 h	14(15.7)	55(30.9)
	Greater than 1 h	48(53.9)	60(33.7)
History travel 7–18 days prior onset of illness	Yes	29(32.6)	16(9.0)
	No	60(67.4)	162(91.0)
Contact history with measles cases	Yes	47(52.8)	45(25.3)
	No	42(47.2)	133(74.7)

### Description of Disease by a Person

Out of the total cases, 5 (5.6%) cases were confirmed by laboratory testing (IgM positive), while the remaining 84 cases had epidemiological linkage with the laboratory-confirmed cases (Table 2). Regarding age-specific attack rate (AR) and case fatality rate (CFR), the highest attack rate and case fatality rate occurred among the age group of 1–4 years (4.27/1000 and 8.3%, respectively) (Table 3). As compared to men, a higher sex-specific attack rate was observed among females (2.04/1000) (Table 3).

**Table 2:** Measles disease attack rate and case fatality rate by age category in Aneded district, Northwest Ethiopia, 2020

Category	Total population	Number of cases	number of deaths	AR per 1000 population	CFR (%)
Sex					
Female	27,482	56	2	2.04	3.57
Male	26,405	33	1	1.25	3.03
Age group					
< 1 year	1678	2	0	1.19	0
1–4 years	5618	24	2	4.27	8.3
5–14 years	15,648	25	1	1.60	4.0
≥ 15 years	30,943	38	0	1.22	0
Kebeles					
Adisgie Yegora	8379	1	0	0.12	0
Amber Town	2597	3	0	1.15	0
Amber Zuria	8782	17	0	1.94	0
Jamma	9894	55	1	5.56	1.82
Malgash	7378	3	0	0.41	0
Tikur adbir	3899	7	2	1.79	28.57
Talak amba	5372	2	0	0.37	0
Wonga nefasam	7586	1	0	0.13	0
Total	53,887	89	3	1.65	3.4

**Table 3:** Characteristics of Measles cases during measles outbreak investigation in Aneded district, northwest Ethiopia, 2020

Variables	Frequency	Percentage
**Case classification**		
Laboratory confirmed (Ig M positive)	5	5.6
Epidemiological linked	84	94.4
**Complication status**		
Not complicated	67	75.3
Complicated	22	24.7
**Type of complication**		
Pneumonia	10	45.4
Diarrhea	7	31.8
Ear infections	5	22.8
**Vitamin A supplementation**		
Not taking	39	43.8
Taking	50	56.2

There were 22 complicated cases [(24.7%) 95% CI; 15.7-34.8%] with histories of pneumonia, diarrhea, and ear infections in 10 (11.2%), 7 (7.9%), and 5 (5.6%) of the patients, respectively. Following a measles diagnosis, vitamin A supplementation was administered to almost half (56.2%) of the cases (Table 2).

### Description of Disease by Place

Eight (or 40%) of the district’s total kebeles experienced the outbreak. The highest attack rate, 5.55/1000, was found in Jamma Kebele, where 55 cases, or 61.8%, were investigated. ‘In Jamma Kebele, there was a one-year interruption to two outreach vaccination sites. In Jamma Kebele, the index case with a history of measles contact and travel was found’. Two (66.7%) of the total deaths were reported to have occurred in Tikur Adbir Kebele (Table 3).

### Description of Disease by the Time

**Fig. 2:**
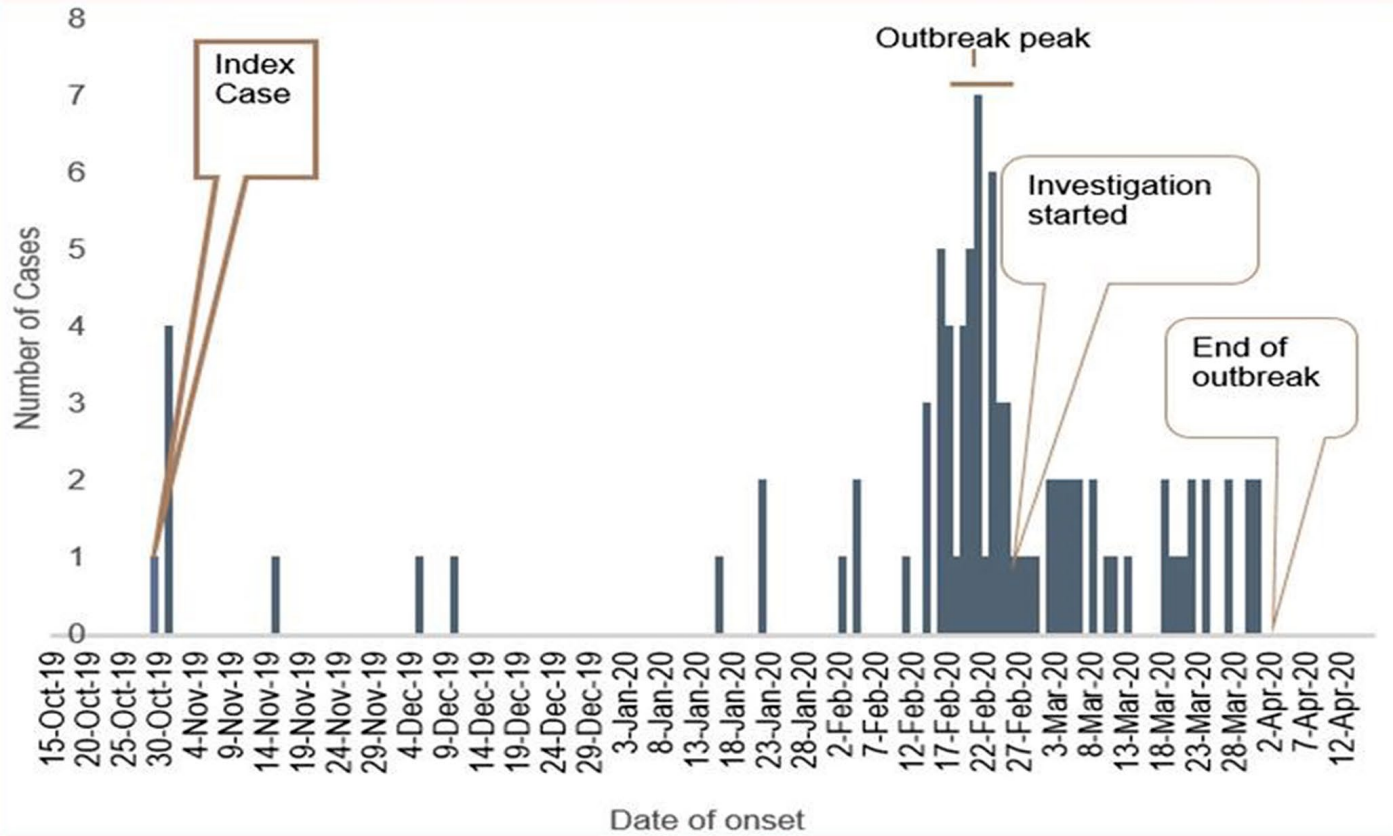
Epi curve shows the distribution of measles cases by date of onset of signs and symptoms in Aneded district, Northwest Ethiopia, 2020

On November 4, 2019, the index case was brought to the health center. Following the index case’s symptoms on October 30, 2019, the outbreak lasted for 155 days, ending on April 1, 2020. March 1, 2020, to February 16, 2020, was the start date of more than half (51.7%) of cases. Eight days (IQR = 3–14) was the median date of instances reported by medical staff or patients visiting hospitals. Approximately forty (44.9%) of the cases were found within 8 days of the onset of symptoms. After the rash had appeared for 48 h, nearly four out of five instances (78.7%) had been identified. The epidemic curve revealed that there was person-to-person transmission. The outbreak investigation was initiated after the outbreak peaked. The outbreak ended on April 2, 2020 (Fig. 2).

### Knowledge of Household Heads Toward Measles Disease

Among the total study participants, 176 (65.9%) said measles was not caused by a virus. More than two-thirds, or 180 (67.4%) of study participants, answered that measles was not communicable. Regarding the measles symptom, 260 (97.4%) of participants reported rash as the symptom of measles. Three-fourths of the 201 (75.3%) study participants said that measles had no medical treatment (Table 4).

**Table 4:** Knowledge of household heads towards measles disease in Aneded district, Northwest Ethiopia, 2020

Knowledge related questions	Possibility response and number participants with percentage	
	Yes	No
Measles is caused by virus	91 (34.1%)	176 (65.9%)
Measles is communicable disease	87 (32.6%)	180 (67.4%)
Measles is vaccine preventable disease	69 (25.8%)	198 (74.2%)
Measles can be transmitted only from person to person	76 (28.5%)	191 (71.5%)
**Symptoms**		
Fever	252 (94.4%)	15 (5.6%)
Rash	260 (97.4%)	7 (2.6%)
Runny nose	178 (66.7%)	89 (33.3%)
Cough	78 (29.2%)	189 (70.8%)
Redness of the eye	98 (36.7%)	169 (63.3%)
Measles has medical treatment	66 (24.7%)	201 (75.3%)
Isolation of patient is measles disease control method	82 (30.7%)	185 (69.3%)
Measles can lead to death	59 (22.1%)	208 (77.9%)

### Attitude of Household Heads Toward Measles Disease

Of the total study participants, one-fourth, or 25.1%, disagreed with the belief that the measles is brought on by supernatural forces. Nearly four-tenths (39.3%) of study participants agree that health professionals prescribed drugs’ aggravate measles disease. Seventy-three (27.3%) of study participants strongly agree that taking vaccines prevents measles disease. Nearly three-tenths (31.1%) of study participants strongly agree that measles isn’t dangerous and therefore vaccination is not really (Table 5).

**Table 5:** Attitude toward measles disease prevention and control of Study participates in Aneded district, Northwest Ethiopia, 2022

Attitude	Alternatives				
	Strongly disagree	Disagree	Uncertain	Agree	Strongly agree
Cause of measles disease is supernatural.	64(24.0%)	67(25.1%)	74(27.7%)	27(10.1%)	35(13.1)
Taking vaccine prevent measles disease	31(11.6%)	69(25.8%)	62(23.2%)	32(12.0%)	73(27.3%)
Health professional prescribed drugs’ aggravate measles disease.	22(8.2%)	49(18.4%)	58(21.7%)	105(39.3%)	33(12.4%)
Special coffee ceremony is the way of management of measles disease	31(11.6%)	24(9.0%)	20(7.5%)	122(45.7%)	70(26.2%)
Taking bath may complicate the disease.	52(19.5%)	26(9.7%)	47(17.6%)	54(20.2%)	88(33.0%)
Shadow/contact complicates the disease.	42(15.7%)	21(7.9%)	38(14.2%)	94(35.2%)	72(27.0%)
I think measles disease is communicable	88(33.3%)	71(26.6%)	36(13.5%)	50(18.7%)	21(7.9%)
Measles isn’t dangerous and therefore vaccination is not really necessary	40(15.0%)	32(12.0%)	37(13.9%)	75(28.1%)	83(31.1%)

### Analytical Study

Multivariable logistic regression revealed that there was a significant association between the measles and factors such as gender [AOR = 2.66; 95% CI 1.38–5.11], age under 5 [AOR = 7.24; 95% CI 2.58–20.31], attitude [AOR = 0.22; 95% CI 0.11–0.42], history of travel 7–18 days prior to illness onset [AOR = 2.94; 95% CI 1,23–7.11], and contact history [AOR = 3.19; 95% CI 1.67–6.10] (Table 6).

**Table 6:** Bivariable and multivariable binary logistic regression analysis of factors associated with measles disease in Aneded district, Northwest Ethiopia, 2020

Variables	Case	Control	COR 95% CI	AOR 95% CI	*P*-value
**Sex**	*N* (%)	*N* (%)			
Male	33(37.1)	97(54.5)	1	1	
female	56(62.9)	81(45.5)	2.03(1.21–3.42)	2.66(1.38–5.11)	0.003
**Age in year**					
< 5	26(29.2)	9(5.1)	8.36(3.60-19.43)	7.24(2.58–20.31)	0.000
5–14	25(28.1)	59(33.1)	1.23(0.68–2.22)	1.35(0.68–2.70)	0.389
≥ 15	38(42.7)	110(61.8)	1	1	
**Knowledgeable**					
Yes	20(22.5)	64(36.0)	0.52(0.29–0.93)	0.76(0.36–1.57)	0.454
No	69(77.5)	114(64.0)	1	1	
**Attitude**					
Favorable	37(41.6)	139(78.1)	0.20(0.12–0.35)	0.22(0.11–0.42)	0.000
Unfavorable	52(58.4)	39(21.9)	1	1	
**Vaccination status**					
Yes	20(22.5)	52(29.2)	1	1	
No	69(77.5)	126(70.8)	0.70(0.39–1.27)	0.68(0.32–1.43)	0.306
**History travel 7–18 days before the onset of illness**					
Yes	29(32.6)	16(9.0)	4.89(2.48–9.64)	2.94(1.23–7.01)	0.015
No	60(67.4)	162(91.0)	1	1	
**Time is taken to reach the nearest health facility**					
1 h	27(30.3)	63(35.4)	1	1	
Less than 1 h	14(15.7)	55(30.9)	0.59(0.28–1.24)	0.47(0.19–1.14)	0.094
Greater than 1 h	48(53.9)	60(33.7)	1.87(1.03–3.36)	1.37(0.65–2.86)	0.407
**Contact history with measles cases**					
Yes	47(52.8)	45(25.3)	3.31(1.93–5.65)	3.19(1.67–6.10)	0.000
No	42(47.2)	133(74.7)	1	1	

### Public Health Interventions

About 56.2% of cases had been taking vitamin A, and all complicated cases were managed accordingly. We have reported our outbreak investigation findings to the district rapid response team based on the recommendation the district health office made and launched the measles vaccination.

## Discussion

The study’s objective was to characterize an outbreak in terms of person, place, and time and identify determinants. It discovered an overall AR of 1.65 per 1,000 people, which is comparable to findings from earlier research conducted in Ethiopia’s Basso Liben district (1.24 per 1,000 population) [21] and another study conducted in the Amhara region of Ethiopia (1.78 per 1000) [22]. The overall AR of this study was lower than the study conducted in Thailand (9.7 per 1000 population) [23], a remote area of the Solomon Islands (123 per 10,000 population) [24], the Dwarahat block of District Almora, Uttarakhand (2.8%) [25], and the South West Shoa Zone of the Oromia Region, Ethiopia (2.34%) [26]. This variation might be due to the difference in living conditions. In the previous studies, the households were more crowded than the households in this study. Overcrowded environments always increase the transmission of the measles virus [27], which could increase the risk of contracting measles. When this study is compared with the study conducted in the Guji zone of the Oromia Region, Ethiopia, 81 per 100,000 [28], Ataq and Habban districts, Shabwah governorate, Yemen, 82 per 100,000 [29], and the Republic of Serbia, 5.8 per 100,000 [30], it was higher. The higher attack rate might be due to late detection and confirmation of the outbreak. Another justification might be due to the increase in the susceptible population, which may have contributed to the spread of the disease faster than predicted.

The age range of 1–4 years old had the highest attack rate (4.27/1000). This result was in line with a study carried out in Uganda [31].There was a disruption at Jamma Keble’s outreach vaccination site [13]. In the age range of 1–4 years, the attack rate may rise due to the accumulation of vulnerable individuals brought on by inadequate vaccination coverage [32].

Overall, 3.4% (95% CI: 1.9–7.9%) of the patients had a fatal outcome. The value gathered was higher when compared with studies conducted in an urban slum of Kaduna Metropolis, Kaduna State, Nigeria 1.3% [33] and the Guji zone of Oromia Region, Ethiopia 0.2% [28]. This discrepancy could be the result of difference in epidemic response. Following the deaths of two newborns, the outbreak under investigation was identified. Patients who did not receive treatment ran the danger of developing complications from the measles, including fatalities [14].

The results of this study, however, were in line with those of a study done in a rural Nigerian area at 6.9% [33] and on the Indo-Myanmar border in Longding District, Arunachal Pradesh, India, at 7% [27]. Parents who hide a sick child from their children increase the complications and case fatality rate of measles [12].

The outbreak lasted 155 days, with 51.7% of cases occurring in February. The population movement and many traditional ceremonies (weddings, religious festivals) during this season produced a favorable situation for measles transmission [35], which may increase the duration of the outbreak. This finding was consistent with studies conducted in Nigeria [36] and Ethiopia [12].

Additionally, this study looked for variables that were linked to the contracting of the measles. It was discovered that the following variables were significantly linked to the infection of the measles virus: being female, being younger than five years old, having a positive attitude toward the prevention and control of the measles, having traveled within the last seven to eighteen days, and having come into contact with cases of the disease. In this investigation, females are 2.6-fold more likely to contract the measles virus due to cultural influence and their role as caregivers, potentially exposing them to sick children. This finding is consistent with the study conducted in Artuma Fursi Woreda, Ethiopia [37]. This finding is different from the study conducted at the Gwagwalada Area Council of the Federal Capital Territory (FCT) of Nigeria, Abuja [3]. The incidence of measles is higher in boys even when vaccination rates are equal for both sexes because females have higher antibody titers and survival durations [38]. On the other hand, greater rates of infection-related death have been noted globally for females of all ages, with a growing sex bias among individuals of reproductive age. This could be attributed to larger doses of exposure, disparities in access to healthcare, or unique immunity in women [38]. In our current study, among male measles cases, nearly four-tenths (39.4%) of males had a vaccination history, whereas among female measles cases, 12.5% of females had a history of vaccination status.

Children under the age of five are seven times more likely to contract the measles virus than older children. This could be because of the district’s low immunization rate, which has raised vulnerability and caused outbreaks. This study’s findings are consistent with those of a study conducted in the Amhara region [35].

Individuals with favorable household heads’ attitudes towards measles disease prevention and control were 78% less likely to contract the virus. Households with a favorable attitude might vaccinate their children. This is congruent with the studies conducted in Kenya [39], Australia [40], and the United States [41].

Travel history, involving 7–18 days of prior exposure to the measles outbreak area, increases the risk of contracting the virus by three times compared to those without such a history. This finding is consistent with the study done in California [42].

Individuals with a contact history with measles patients 2–3 weeks before their current infection have three-fold increased odds of contracting the measles virus. Measles is a highly contagious disease transmitted through respiratory droplets or airborne spread. So, a history of contact can increase virus transmission to susceptible hosts [43].This result is consistent with the study conducted in Ethiopia [22].

## Conclusion

The measles outbreak with higher attack and case fatality rates has been influenced by factors such as the attitude of household heads, age, sex, and contact and travel history. To reduce attack and case fatality rates, early detection and response through passive and active surveillance should be strengthened. Inducing favorable attitudes in household heads and caregivers toward measles disease prevention and control should be strengthened to reduce contracting the measles virus. Those individuals who have travel and contact histories should be traced as early as possible to reduce the spread of the outbreak.

### Limitations

Mothers or caregivers kept the ill child hidden from outsiders in their homes, often for days at a time. If an outbreak is discovered too late, individuals may recover on their own, or all community deaths due to the measles may go unrecorded. They may lead to a decrease in the attack rates of measles cases as well as the case fatality rate. The small sample size may make the generalizability less reliable.

## Data Availability

The data sets generated during the current study are available from the corresponding author on reasonable request.

## Abbreviations

AOR: Adjusted odds ratio
CI: Confidence interval
HIV: Human immune deficiency virus
WHO: World health organization
